# Verification and Validation of a Four-Gene Panel as a Prognostic Indicator in Triple Negative Breast Cancer

**DOI:** 10.3389/fonc.2022.821334

**Published:** 2022-03-21

**Authors:** Mamta Pariyar, Rick F. Thorne, Rodney J. Scott, Kelly A. Avery-Kiejda

**Affiliations:** ^1^ School of Biomedical Sciences and Pharmacy, College of Health, Medicine and Wellbeing, University of Newcastle, Callaghan, NSW, Australia; ^2^ Hunter Medical Research Institute, New Lambton Heights, NSW, Australia; ^3^ Translational Research Institute, Henan Provincial People’s Hospital, Academy of Medical Science, Zhengzhou University, Zhengzhou, China; ^4^ NSW Health Pathology, John Hunter Hospital, New Lambton Heights, NSW, Australia

**Keywords:** triple negative breast cancer (TNBC), metastasis, gene expression, ddPCR, prognostic

## Abstract

Triple negative breast cancer (TNBC) is a highly aggressive subtype with a high rate of metastasis, early distant recurrence and resistance to therapy leading to worse survival than other breast cancer subtypes. There are no well-established biomarkers that can determine women who will do better and those who are likely to have poorer outcomes with TNBC, nor are there targeted therapies. Thus, the identification of prognostic and/or predictive biomarkers will enable tailored therapies based on their likelihood of disease outcomes and may prevent over- and under-diagnosis. Previous studies from our laboratory have identified four genes (ANP32E, DSC2, ANKRD30A and IL6ST/gp130) that are specific to TNBC and were associated with lymph node metastasis (LNmets), the earliest indicator of tumor progression *via* distal spread. This study aimed to validate these findings using absolute quantitation by digital droplet PCR (ddPCR) and to determine relationships with clinicopathological features and survival. Our analysis confirmed all four genes displayed significant expression differences between TNBC cases and non-TNBC cases. Moreover, low IL6ST expression was significantly associated with grade 3 disease, hormone receptor negativity and earlier age at diagnosis; low ANKRD30A expression was associated with tumor size; and high ANP32E expression was significantly associated with grade and the number of positive lymph nodes. Individually, three of the four genes were associated with relapse-free survival in TNBC and in combination, all four genes were significantly associated with TNBC survival, but not in hormone receptor-positive cases. Collectively our results suggest that the four genes may have utility in TNBC prognostication.

## Introduction

Triple negative breast cancer (TNBC) is one of the most aggressive subtypes due to it not being amenable to targeted therapies including Tamoxifen and Herceptin (trastuzumab) and it is associated with rapid metastasis, higher risk of recurrence and poorer survival outcomes, when compared to receptor positive breast cancer subtypes (1, 2). Because of the lack of targeted treatment, the current treatment options are limited to chemotherapy and surgery. There are no well-established prognostic biomarkers in TNBC that can be used in disease prognosis. Therefore, identification of prognostic biomarkers to improve treatment regimens and that can potentially be targets for therapy in this breast cancer subtype are urgently required.

Previous studies from our laboratory have identified four genes that are differentially expressed in invasive ductal carcinoma (IDCs) compared to normal adjacent tissues (NATs) in TNBC as well as being differentially expressed in TNBC compared to non-TNBC. These results were validated in two independent cohorts, including a large cohort sourced from The Cancer Genome Atlas (TCGA) using the same method (cDNA microarrays) (3). Ankyrin repeat domain 30A (*ANKRD30A*) and interleukin 6 signal transducer (*IL6ST*) were downregulated in TNBC compared to non-TNBC, whereas desmocollin-2 (*DSC2*) and acidic nuclear phosphoprotein 32 family member E (*ANP32E*) were upregulated in TNBC compared to non-TNBC. *ANKRD30A* is a breast differentiation antigen responsible for protein-protein interactions and other cellular functions (4). *IL6ST* also known as glycoprotein 130 (gp130) is a signal transducer for the interleukin family of cytokines such as IL6, CNTF, LIF and OSM; and an activator of JAK/STAT and MAPK/PI3K/ERK signaling pathways (5). *DSC2* is one of the main components of desmosomes, which aid in cell-cell attachments as well as play a key role in cell growth and apoptosis (6). *ANP32E* is a histone chaperone that has the ability to strip H2A.Z away from DNA, allowing chromatin remodeling and thus altering gene expression (7). Although some of these genes have been implicated in other types of breast cancer, the relationship of these genes to prognosis in TNBC is currently unknown. Moreover, our previous results need to be verified using a different method in order to move these results forward to the clinic.

Amplification by digital droplet PCR (ddPCR) offers several advantages over conventional qRT-PCR; it can be used to calculate the absolute concentration of cDNA in a sample without the need for any standards, as the cDNA within a sample is partitioned into thousands of droplets, amplified and counted directly by Poisson statistics. Because of the sample partitioning and endpoint quantitation used in ddPCR, PCR amplification is independent of reaction efficiency as well as being less susceptible to Taqman polymerase inhibitors compared to that in qPCR in which PCR amplification is dependent on the concentration of inhibitors in the entire sample. Thus ddPCR provides accurate, precise and reproducible data (8, 9) and also can be used for low concentration samples with increased precision (10). Moreover, ddPCR can be utilized to perform multiplexing to detect more than one target in the reaction, reducing the time and cost of the experiment (11).

The aim of this study was to verify the differential expression of *ANKRD30A*, *IL6ST*, *DSC2* and *ANP32E* in TNBC compared to NATs and between TNBC and non-TNBC using an independent method (ddPCR) and to define their relationship with clinicopathological features and survival outcomes. We have shown that these genes were significantly different between TNBC cases and non-TNBC cases. Individually, three of the four genes were associated with relapse-free survival in TNBC and when combined, the four genes were significantly associated with survival in TNBC, but not in hormone receptor-positive cases. Thus, *ANKRD30A*, *IL6ST*, *DSC2* and *ANP32E* may represent novel prognostic markers for the TNBC subtype.

## Materials and Methods

### Cohorts

Two cohorts were used in this study. The first cohort consisted of a total of 28 invasive ductal carcinomas of the TNBC subtype, with 13 matched LNmets and 2 unmatched LNmets as well as 8 matched normal adjacent tissue (NAT) and 1 unmatched NAT. All samples were formalin fixed paraffin embedded (FFPE) and obtained by 1.5mm punch biopsy from the archives of NSW Health Pathology, John Hunter Hospital, Newcastle, Australia. This cohort has been described previously (3). Areas of IDC, LNmet and NAT were identified and confirmed by a pathologist. The clinical characteristics of the patients used in this study are shown in **Table 1**.

**Table 1:** Clinical characteristics of 28 TNBC samples of the first cohort.

Variable	n (%)
**Age (years)**	
Median (Range)	55 (36-84)
<40	2 (7.1%)
40-50	9 (32.1%)
>50	17 (60.7%)
**Grade**	
1 or 2	5 (17.8%)
3	23 (82.1%)
**Tumour size (mm)**	
median	28
≤28	15 (53.5%)
>28	13 (46.4%)
**No. of positive lymph nodes**	
0	13 (46.4%)
1-3	11 (39.2%)
>3	3 (10.7%)
	LN status of 1 case is unavailable.

A second cohort with a total of 13 TNBCs and 105 non-TNBCs for comparisons with a non-TNBC cohort. The samples were fresh frozen IDC and were provided by the Australian Breast Cancer Tissue Bank (Westmead, NSW, Australia), which have been previously described (12). The cohort characteristics are described in **Table 2**.

**Table 2:** Clinical characteristics of 118 (TNBC and non-TNBC) samples of the second cohort.

Variable	n (%)
**Age years**	
Median (Range)	56 (28-90)
<40	12 (10.1%)
40-50	36 (30.5%)
>50	70 (59.3%)
**Grade**	
1 or 2	59 (50%)
3	59 (50%)
**Tumour size (mm)**	
median	25
≤25	65 (55.0%)
>25	53 (44.9%)
**No. of positive lymph nodes**	
0	52 (44.0%)
1-3	46 (38.9%)
>3	20 (16.9%)
**ER**	
Positive	93 (78.8%)
Negative	25 (21.1%)
**PR**	
Positive	86 (72.8%)
Negative	32 (27.1%)
**HER2**	
Positive	18 (15.2%)
Negative	99 (83.8%)
	HER2 status of 1 case is unavailable
**TNBC**	
Yes	13 (11.0%)
No	105 (88.9%)

This study complies with the Helsinki Declaration with ethical approval from the Hunter New England Human Research Ethics Committee (Approval number: 09/05/20/5.02). In accordance with the National Statement on Ethical Conduct in Research Involving Humans, a waiver of consent was granted for cases from NSW Health Pathology, whilst all other cases have consented to their tissue and clinical information being used for research.

### RNA Extraction and Quantification

RNA extraction of the whole biopsy samples was previously described (13). All samples were stored at -80°C. These were quantitated before cDNA synthesis using the Qubit™ RNA BR (broad range) Assay Kit. The extracted FFPE DNA stored at -20°C was quantitated using the Qubit^®^ dsDNA HS Assay Kit.

### Reverse Transcription

Either 75ng RNA from fresh frozen tissues or 125 ng RNA from FFPE tissues was used for cDNA synthesis, the latter amount increased to counter the highly degraded nature of the RNA in FFPE samples. cDNA synthesis was performed as using the High-Capacity cDNA Reverse Transcription Kit (Life Technologies, Mulgrave, VIC, Australia) to generate complementary DNA (cDNA) according to the manufacturers’ instructions.

### Digital Droplet PCR (ddPCR)

A total reaction volume of 25 μl was prepared according to the manufacturers’ instructions (Bio-Rad). The amount of cDNA added to the PCR reaction depended on whether it was extracted from fresh frozen or FFPE tissues, due to the differing amplification efficiencies: 12.5 ng cDNA equivalent to RNA input from FFPE tissues was used in the reaction; while 3.75 ng cDNA equivalent to RNA input that had been reverse transcribed from fresh frozen tissues was used. TaqMan Gene Expression Assays (Life Technologies) for *ANKRD30A* (Hs00369567_m1), *IL6ST* (Hs00174360_m1), *ANP32E* (Hs01064731_m1) and *DSC2* (Hs00951428_m1) were used for digital droplet PCR. Droplets were generated in an Automated Droplet QX200 Generator (1864101, Bio-Rad) according to the manufacturers’ instructions. PCR amplification of the cDNA within the droplets was performed using the C1000 thermal cycler (Bio-Rad) according to the manufacturers’ instructions.

After PCR amplification of the target cDNA within the droplets, the sample plate was placed in the droplet reader (QX200 Droplet Reader, 1864003, Bio-Rad) which counts each droplet individually using a fluorescent detection system which is set to detect FAM or HEX/VIC and classifies them as positive or negative droplets based on endpoint fluorescent amplitude. Positive droplets containing at least one copy of the target cDNA molecule have an increased fluorescence compared to negative droplets. The number of positive and negative droplets read by droplet reader was then used by Quantasoft software (Bio-Rad) to calculate the absolute quantity of DNA per sample in copies/μl where it first determines the fraction of positive droplets and after combination with a Poisson algorithm, provides the original concentration of the target template. Based on Poisson statistics, the average copies (of target) per droplet (CPD) was calculated as: *CPD* = −ln(1−*p*); where p = fraction of positive droplet. CPD can then be converted into the concentration of target (copies/μl) in the initial sample as shown below:


$$Concentration\;(copies/\mu \text{L})=CPD/V^{droplet};$$

where V^droplet^ is the average droplet volume (*μ*L).

### Statistical Analysis

All statistical analysis was performed using GraphPad Prism 7 (San Diego, California, USA). The normality of the distribution was tested using the D’Agostino & Pearson normality test. As some of the groups were not normally distributed, a Kruskal-Wallis test was used to determine if differences in the expression of *DSC2*, *ANP32E* and *IL6ST* were statistically significant between unmatched groups. A two-tailed Mann Whitney test was used to assess if there was a statistically significant difference in the expression of *DSC2*, *ANP32E, IL6ST* and *ANKRD30A* between two groups. To assess if the differential expression was associated with clinical features including age, tumor size, grade and lymph node positivity, a chi-squared test was used. A p value ≤ 0.05 was considered to be statistically significant.

### Kaplan-Meier (KM) Plotter Database Analysis

KM plotter is a publicly available online database with gene expression and survival information data downloaded from Gene Expression Omnibus (GEO), European genome-phenome Archive (EGA) and The Cancer Genome Atlas (TCGA). This database was used to perform relapse free survival analysis in TNBC and non-TNBC cases. Each gene of interest was entered into the database to obtain KM survival curves plots and number at risk. The following probe IDs were used for this analysis: *ANKRD30A* (223864_at), *IL6ST* (204863_s_at, 204864_s_at, 211000_s_at, 212195_at, 212196_at), *DSC2* (204750_s_at, 204751_x_at, 226817_at) and *ANP32E* (208103_s_at, 221505_at). A total of 255 TNBC cases in the database were available for RFS analysis on *ANP32E* and IL6ST, while only 161 TNBC cases were available for RFS analysis with the *DSC2* and *ANKRD30A* microarray probes. No gene expression microarray data for *DSC2* and *ANKRD30A* were available in the rest of the 94 TNBC samples. The automatically generated best cut-off (more accurate than median) was chosen to classify the expression of genes into high and low values. The best cut-off provided in the KM plotter is the best performing cut-off with the most statistically significant p-value (Cox regression analysis) from all the possible cut-offs computed automatically by the database between the lower and upper quartiles. The publicly available microarray datasets including E-MTAB-365, E-TABM-43 and GSE (Gene Expression Omnibus Series) in the software were selected to generate the Kaplan-Meier plots in this study. A log rank p-value of 0.05 was considered statistically significant. The hazard ratio, 95% confidence interval and number at risk were obtained using the database.

## Results

### Verification of Differential mRNA Expression of ANP32E, DSC2, IL6ST and ANKRD30A Between Normal and Tumour Samples by ddPCR

**Figure 1:**
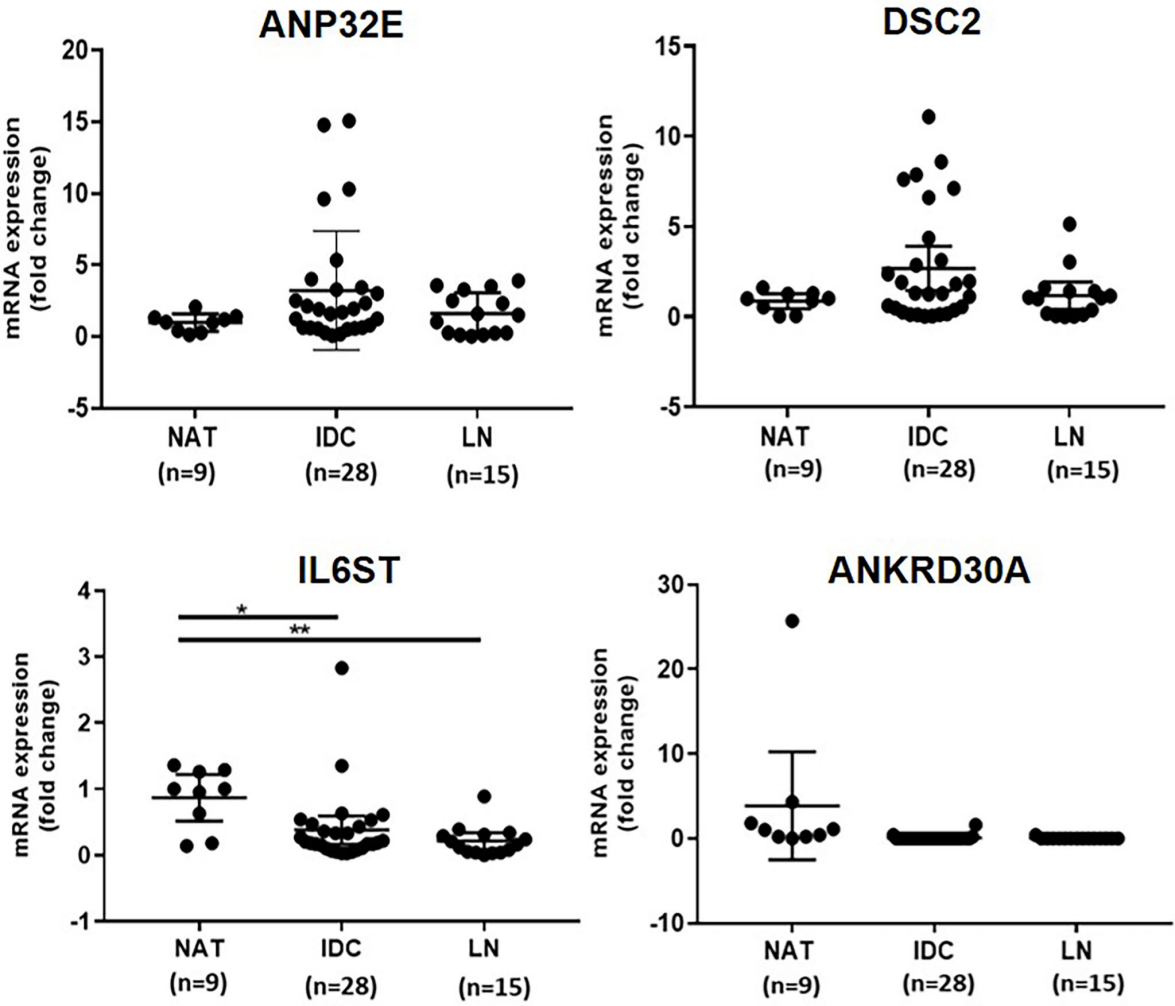
Verification of differential expression of *ANP32E, DSC2, IL6ST* and *ANKRD30A* genes at the mRNA level in tumour compared to normal tissue in the first cohort. The mRNA expression of *ANP32E, DSC2, IL6ST* and *ANKRD30A* genes obtained as copies per μl using ddPCR. Results show the fold change expression of four genes in IDCs and LNmets compared to NATs. Values are presented as the median ± interquartile range. A Kruskal-Wallis test followed by Dunn’s test for multiple comparisons was performed to determine the statistical significance of the expression. A p value ≤ 0.05 was considered significant. Asterisks in the figure represents statistical significance (*p ≤ 0.05; **p ≤ 0.01). NAT, Normal adjacent tissue; IDC, Invasive ductal carcinoma and LNmet, Lymph node metastasis.

The mRNA expression of *ANP32E*, *DSC2*, *IL6ST* and *ANKRD30A* was quantified using ddPCR to verify the differential mRNA expression of these genes in IDC (n = 28) compared to NAT (n = 9) as well as LNmets (n = 15). This was performed in the first cohort, which was previously used for gene expression analysis in a recent publication by our group (3). The sample number used in this study varied from the numbers in the previous study as some samples were excluded due to the low RNA amount. In concordance with our previous findings (3), the expression of *IL6ST* was significantly downregulated in IDCs (median fold change = -0.2) and LNmets (median fold change = -0.2) compared to NATs (p=0.0171 and 0.0020, respectively). The expression of *DSC2* (median fold change = 1.3) and *ANP32E* (median fold change = 1.8) were upregulated in IDCs compared to NATs, however, the increase in expression was not statistically significant (p = 0.4537, p = 0.1743, respectively) (**Figure 1**). Additionally, the expression of these genes was increased in LNmets when compared to NAT (median fold change for *ANP32E* = 1.5), but this difference was not significant (p ≥ 0.999 and 0.9864, respectively). *ANKRD30A* showed very low to no expression in IDCs and LNmets compared to NATs and was undetectable in the majority of samples (**Figure 1**).

### Verification of Differential mRNA Expression of ANP32E, DSC2, IL6ST and ANKRD30A Between TNBC and Non-TNBC by ddPCR

**Figure 2:**
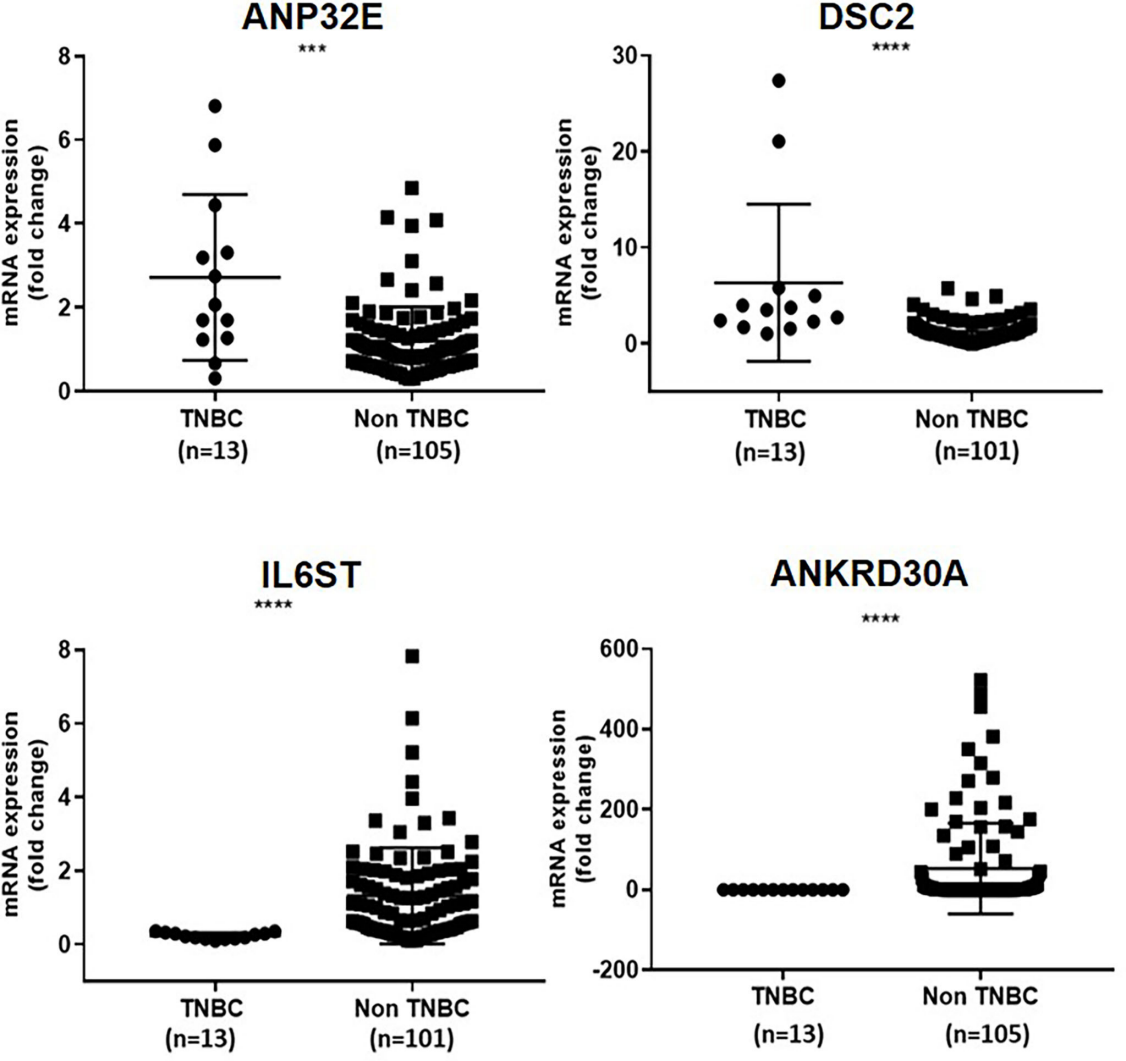
Verification of differential expression of *ANP32E, DSC2, IL6ST* and *ANKRD30A* genes in TNBC compared to non-TNBC at the mRNA level in the second cohort. The mRNA expression of *ANP32E, DSC2, IL6ST* and *ANKRD30A* genes obtained as copies per μl using ddPCR. Results are shown as fold change expression of target genes in TNBC compared to non-TNBC. Values are presented as the median ± interquartile range. A Mann Whitney test was used to determine the statistical significance of the expression in TNBC. A p value ≤ 0.05 was considered significant. Asterisks in the figure represent statistical significance (***p ≤ 0.001, ****p ≤ 0.0001).

Next, the differential expression of ANP32E, DSC2, IL6ST and ANKRD30A in TNBC compared to non-TNBC was verified using digital droplet PCR. For this analysis, 13 TNBC and 105 non-TNBC samples (second cohort) were used. This was to confirm the differential expression of these genes identified in our previous study at the mRNA level using a different method. In concordance with our previous finding (3), ANP32E (median fold change = 2.1) and DSC2 (median fold change = 3.5) were significantly upregulated in TNBC compared to non-TNBC (p = 0.0009, p<0.0001, respectively), whereas IL6ST (median fold change = -0.2) and ANKRD30A (median fold change = 0.00) were significantly downregulated in TNBC compared to non-TNBC (p <0.0001, p < 0.0001, respectively) (**Figure 2**).

### Association of mRNA Expression of ANP32E, DSC2 and IL6ST With Clinicopathological Features

To assess whether the mRNA expression of *ANP32E*, *DSC2* and *IL6ST* in TNBC were correlated with clinicopathological features, a chi-square test was performed for all genes except *ANKRD30A* (due to the low number of samples with detectable expression) in relation to age, grade, tumor size, and number of positive lymph nodes. The samples were divided into high (n = 14), and low (n = 14) mRNA expression based on the median expression of each gene within the first cohort. However, the differential mRNA expression of *DSC2*, *ANP32E* and *IL6ST* genes showed no correlation with the clinical characteristics in the first cohort (**Table 3**).

**Table 3:** Association of ANP32E, DSC2 and IL6ST mRNA expression with clinicopathological features in TNBC cases from the first cohort.

Characteristic	ANP32E				DSC2				IL6ST			
	Total	High	Low	P-value	Total	High	Low	P-value	Total	High	Low	P-value
	n=28	n=14 (50%)	n=14 (50%)		n=28	n=14 (50%)	n=14 (50%)		n=28	n=14 (50%)	n=14 (50%)	
**Age (years)**												
<40	2	1 (7)	1 (7)	0.466	2	0 (0)	2 (14)	0.338	2	1 (7)	1 (7)	0.919
40-50	9	6 (43)	3 (21)		9	5 (36)	4 (29)		9	4 (29)	5 (36)	
>50	17	7 (50)	10 (71)		17	9 (64)	8 (57)		17	9 (64)	8 (57)	
**Grade**												
1 or 2	4	1 (7)	3 (21)	0.280	4	2 (14)	2 (14)	>0.99	4	2 (14)	2 (14)	>0.99
3	24	13 (93)	11 (79)		24	12 (86)	12 (86)		24	12 (86)	12 (86)	
**Tumour size (mm), median = 25**												
≤25	13	5 (36)	8 (57)	0.256	13	7 (50)	6 (43)	0.705	13	6 (43)	7 (50)	0.705
>25	15	9 (64)	6 (43)		15	7 (50)	8 (57)		15	8 (57)	7 (50)	
**No. of +ve LNs**												
0	13	8 (57)	5 (36)	0.583	13	7 (50)	6 (43)	0.363	13	7 (50)	6 (43)	0.145
1-3	11	5 (36)	6 (43)		11	5 (36)	6 (43)		11	7 (50)	4 (29)	
>3	3	1 (7)	2 (14)		3	2 (14)	0 (0)		3	0 (0)	3 (21)	

Lymph node status of 1 case is unavailable.

Statistical analyses based on chi squared test. A p value of ≤ 0.05 was considered significant.

In the second cohort, which contained both receptor positive and negative IDC cases, a chi-square test was performed to determine whether the high or low mRNA expression of the four genes was associated with age, grade, tumor size, number of positive lymph nodes, hormone receptor positivity and TNBC status. *ANP32E* was significantly associated with grade (p = 0.0017), number of positive lymph nodes (p = 0.0304); and ER, PR and TNBC status (p = 0.0282, 0.0384 and 0.0081, respectively). *DSC2* expression was significantly associated with ER, PR and TNBC status (p = 0.001, 0.018 and 0.001, respectively). *IL6ST* expression showed a significant association with age (p = 0.05), tumor grade (p < 0.0001) as well as with ER, PR, HER2 and TNBC status (p < 0.0001, < 0.0001, 0.0033 and 0.0001, respectively). *ANKRD30A* expression was significantly associated with tumor size, ER, PR and TNBC status (p = 0.0161, 0.0007, 0.0009, 0.0012, respectively) (**Table 4**).

**Table 4:** Association of *ANP32E, DSC2, IL6ST* and *ANKRD30A* mRNA expression with clinicopathological features in breast cancer cases from the second cohort.

Characteristics	ANP32E				DSC2				IL6ST				ANKRD30A			
	Total	High	Low	P-value	Total	High	Low	P- value	Total	High	Low	P-value	Total	High	Low	P-value
	n=118	n=59 (50%)	n=59 (50%)		n=114	n=57 (50%)	n=57 (50%)		n=114	n=57 (50%)	n=57 (50%)		n=118	n=59 (50%)	n=59 (50%)	
**Age (years)**																
<40	12	5 (8)	7 (12)	0.458	12	5 (9)	7 (12)	0.348	12	2 (4)	10 (18)	**0.05**	12	4 (7)	8 (14)	0.260
40-50	36	21 (36)	15 (25)		35	21 (37)	14 (25)		34	18 (32)	16 (28)		36	16 (27)	20 (34)	
>50	70	33 (56)	37 (63)		67	31 (54)	36 (63)		68	37 (65)	31 (54)		70	39 (66)	31 (53)	
**Grade**																
1 or 2	59	21 (36)	38 (64)	**0.002**	58	24 (42)	34 (60)	0.061	57	40 (70)	17 (30)	**<0.000**	59	34 (58)	25 (42)	0.098
3	59	38 (64)	21 (36)		56	33 (58)	23 (40)		57	17 (30)	40 (70)		59	25 (42)	34 (58)	
**Tumour size (mm), median = 25**																
≤25	65	28 (47)	37 (63)	0.097	62	31 (54)	31 (54)	0.999	62	34 (60)	28(49)	0.259	65	39 (66)	26 (44)	**0.016**
>25	53	31 (53)	22 (37)		52	26 (46)	26 (46)		52	23 (40)	29 (51)		53	20 (34)	33 (56)	
**No. of +ve lymph nodes**																
0	52	21 (36)	31 (53)	**0.030**	49	23 (40)	26 (46)	0.486	49	25 (44)	24 (42)	0.116	52	26 (44)	26 (44)	0.866
1-3	46	30 (51)	16 (27)		46	26 (46)	20 (35)		45	26 (46)	19 (33)		46	24 (41)	22 (37)	
>3	20	8 (14)	12 (20)		19	8 (14)	11 (19)		20	6 (11)	14 (25)		20	9 (15)	11 (19)	
**ER**																
positive	93	44 (75)	49 (83)	**0.028**	90	38 (67)	52 (91)	**0.001**	89	57 (100)	32 (56)	**<0.000**	93	54 (92)	39 (66)	**0.000**
Negative	25	18 (31)	7 (12)		24	19 (33)	5 (9)		25	0 (0)	25 (44)		25	5 (8)	20 (34)	
**PR**																
positive	86	38 (64)	48 (81)	**0.038**	85	37 (65)	48 (84)	**0.018**	83	53 (93)	30 (53)	**<0.000**	86	51 (86)	35 (59)	**0.000**
negative	32	21 (36)	11 (19)		29	20 (35)	9 (16)		31	4 (7)	27 (47)		32	8 (14)	24 (41)	
**HER2**																
Positive	18	10 (17)	8 (14)	0.636	18	10 (18)	8 (14)	0.607	17	3 (5)	14 (25)	**0.003**	18	7 (12)	11 (19)	0.324
Negative	99	49 (83)	50 (85)		96	47 (82)	49 (84)		96	54 (95)	42 (74)		99	51 (86)	48 (81)	
**TNBC**																
Yes	13	11 (19)	2 (3)	**0.008**	13	12 (21)	1 (2)	**0.001**	13	0 (0)	13 (23)	**0.000**	13	1 (2)	12 (20)	**0.001**
NO	105	48 (81)	57 (97)		101	45 (79)	56 (98)		101	57 (100)	44 (77)		105	58 (98)	47 (80)	

HER2 status of 1 case is unavailable.

Statistical analyses based on chi squared test. A p value of ≤ 0.05 was considered significant. The values in bold are significant (p < 0.05).

### Association of mRNA Expression of ANP32E, DSC2, IL6ST and ANKRD30A With Survival

**Figure 3:**
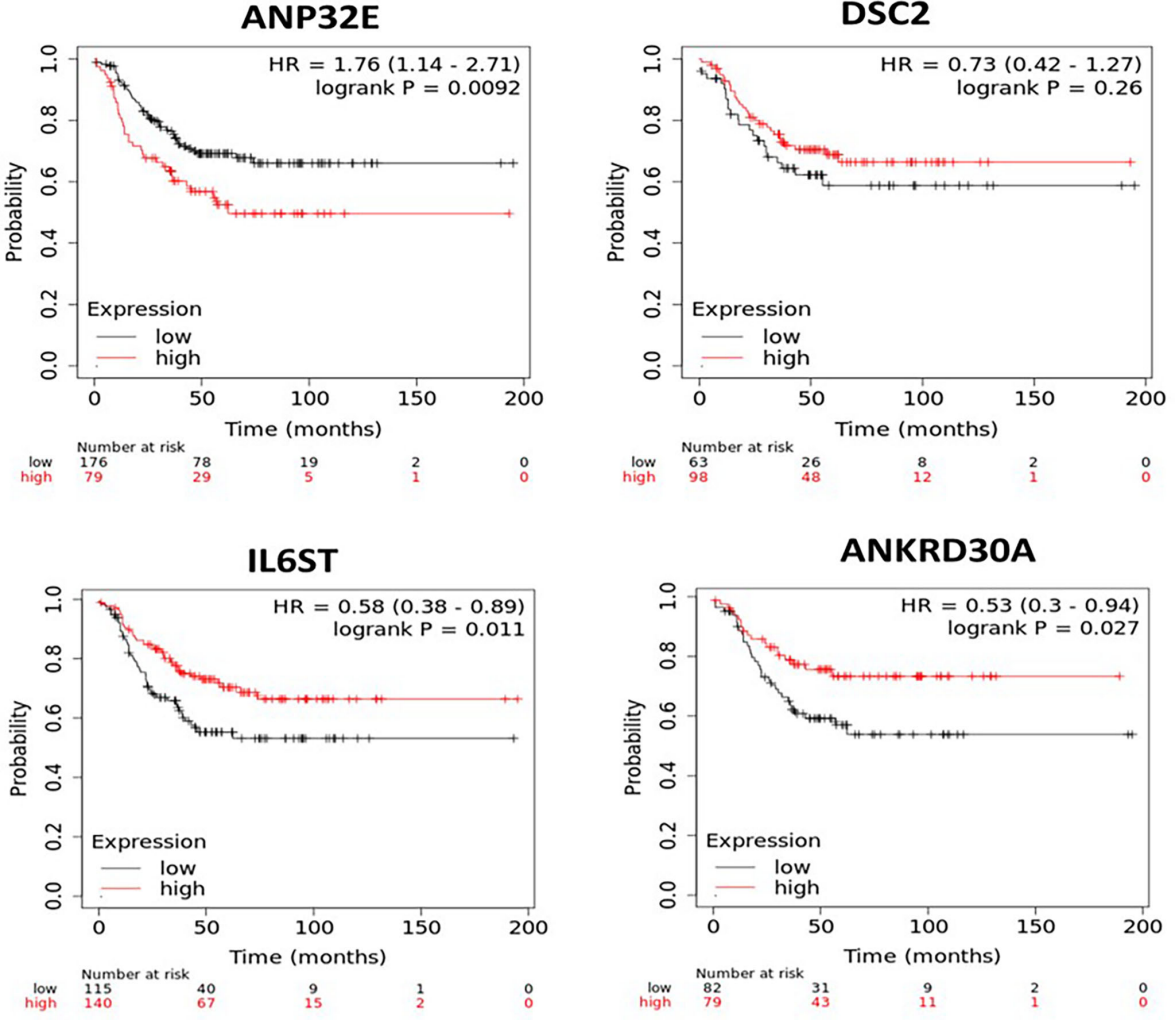
Kaplan-Meier survival curves for patients with high or low mRNA expression of *ANP32E, DSC2, IL6ST* and *ANKRD30A* in TNBC samples. The survival analysis was performed in KM plotter using publicly available TNBC microarray data that is available within the database. TNBC samples in the KM plotter database were divided into high or low expression groups based on auto best cut-off expression value of each gene and compared by Kaplan-Meier survival analysis. A Log-rank p-value ≤ 0.05 was considered significant. HR, hazard ratio. The red curve represents high expression, and the black curve represents low expression of the indicated gene.

Next, the impact of each of the four genes on survival was assessed after segmenting cases into high and low mRNA expression. Due to the low number of TNBC samples in the first and second cohorts, relapse-free survival (RFS) analysis was performed on the four genes in a larger cohort of TNBC cases using the KM plotter online database, which contains expression values from publicly available microarray data. A total of 255 TNBC cases in the database were available for RFS analysis on ANP32E and IL6ST, while only 161 TNBC cases were available for RFS analysis with the DSC2 and ANKRD30A microarray probes. No gene expression microarray data for DSC2 and ANKRD30A were available in the other 94 TNBC samples. High and low expression of these genes were split based on the automatically generated best cut-off value as described the methods. All the microarray probes that were specifically related to the gene of interest were selected. High expression of ANP32E (p = 0.0092) was significantly associated with decreased RFS while the high expression of DSC2 (p = 0.26) showed a non-significant trend of increased RFS. Low expression of IL6ST (p = 0.011) and ANKRD30A (p = 0.027) was significantly associated with decreased RFS (**Figure 3**).

**Figure 4:**
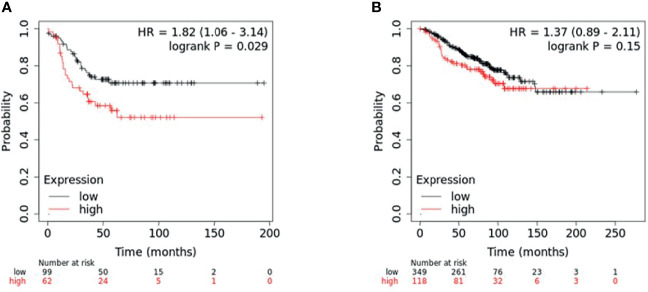
KM plotter survival analysis for patients with differential expression of four-gene panel including *ANP32E, DSC2, IL6ST*, and *ANKRD30A*. Survival analysis was performed in KM plotter using publicly available microarray data that is available within the database. **(A)** TNBC samples (n = 161) and **(B)** non-TNBC (n = 467) were divided into high or low expression groups based on the median expression of the four-gene panel (with inverted expression of the two low expressed genes: ANKRD30A and IL6ST and not inverted for the two highly expressed genes: ANP32E and DSC2) and compared by Kaplan-Meier survival analysis. A Log-rank p-value ≤ 0.05 was considered significant. HR, hazard ratio. The red curve represents high expression, and the black curve represents low expression.

To determine if the combined expression of the four gene panel was associated with survival, relapse free survival analysis of the four gene panel including ANKRD30A, IL6ST, ANP32E and DSC2 was performed using the KM plotter database in both TNBC and non-TNBC samples. For this, the mean expression of the four genes was selected for survival curve analysis with inverted expression of the two low expressed genes: ANKRD30A and IL6ST and not inverted for the two highly expressed genes: ANP32E and DSC2 in both TNBC and non-TNBC samples. A total of 161 TNBC cases and 467 non-TNBC cases was available for four gene panel survival analysis in the KM plotter database. High expression of the four gene panel was significantly associated with low RFS compared to its low expression in TNBC. In contrast, the differential expression of the 4-gene panel in non-TNBC cases (ER+/PR+/HER2+-) was not significantly associated with RFS (**Figure 4**).

**Figure 5:**
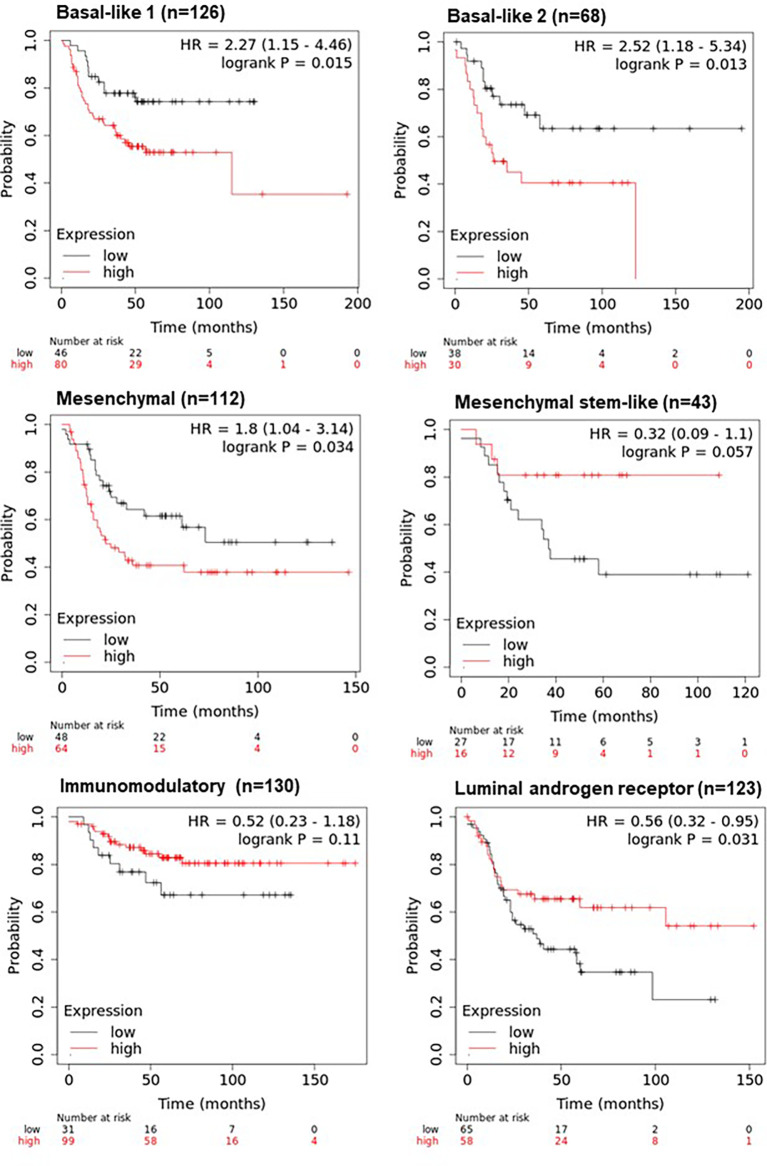
KM plotter survival analysis of TNBC subtypes using the four-gene panel including *ANP32E*, *DSC2*, *IL6ST*, and *ANKRD30A*. Survival analysis was performed in KM plotter using publicly available microarray data that is available within the database according to the TNBC subtypes of basal-like 1, basal-like 2, mesenchymal, mesenchymal stem-like, immunomodulatory and the luminal androgen receptor subtype. Cases were divided into high or low expression groups based on the median expression of the four-gene panel (with inverted expression of the two low expressed genes: ANKRD30A and IL6ST and not inverted for the two highly expressed genes: ANP32E and DSC2) and compared by Kaplan-Meier survival analysis. A Log-rank p-value ≤ 0.05 was considered significant. HR, hazard ratio. The red curve represents high expression, and the black curve represents low expression.

To determine if the combined expression four gene panel was associated with survival, relapse free survival analysis was performed on the 6 TNBC subtypes (14). The four gene panel was associated with worse RFS (when compared to tumours in the low expression group), in the basal-like 1 and 2 (BL1, BL2) subtypes (HR=2.27, p=0.015; HR=2.52, p=0.013 respectively) and the mesenchymal subtype (HR=1.8; p=0.034). In contrast, this four gene panel was associated with better RFS in the luminal androgen receptor (LAR) subtype (HR=0.56, p=0.031) (**Figure 5**). Taken together, these results suggest that the four gene panel can predict distinct survival outcomes amongst the distinct TNBC subytpes, implying that this signature would be useful for most TNBC cases. However, it should be noted that the number of cases in these survival analyses is low, particularly in the mesenchymal stem-like and basal-like 2 subgroups, and should be interpreted with caution.

## Discussion

TNBC is a highly aggressive subtype of breast cancer with decreased survival rates compared to other subtypes of breast cancer (1, 2). Because of the absence of well-established prognostic and predictive biomarkers, there are no targeted treatment options available to improve patient survival. Previous studies from our laboratory have indicated that *ANKRD30A, IL6ST, ANP32E* and *DSC2* are differentially expressed in TNBC when compared to receptor positive disease, using cDNA microarrays. In this study, the expression of *ANKRD30A*, *IL6ST*, *ANP32E* and *DSC2* were analyzed using ddPCR to verify these results and to determine whether their expression was associated with clinical features and survival.

*IL6ST/gp130* was significantly downregulated in IDCs and LNmets compared to NATs as well as in TNBC compared to non-TNBCs, verifying the results of our previous study (3). Additionally, its low expression level was significantly associated with grade 3 disease, hormone receptor negativity, earlier age at diagnosis and worse relapse free survival. Taken together, this implies that its downregulation may increase the aggressiveness of the disease and that its high expression is a marker of good prognosis in TNBC. Similar to this study, *IL6ST* has been shown to be downregulated in basal-like breast cancer compared to luminal A/B breast cancer subtypes and its lower expression was associated with poor overall survival in TNBC patients (15, 16). The loss of *IL6ST* is involved in pathways related to lymphovascular invasion in breast cancer patients (17) and its function has been associated with other physiologies such as myocardial and hematological development where embryos of mice deficient for *IL6ST* gradually die between 12.5 days post-coitum (18). *IL6ST* is known to be involved in the JAK/STAT pathway (19, 20) including the activation of *STAT3* and *STAT1*, as well as the PI3K/AKT and RAS/MAPK pathways. Therefore, its loss in TNBC may disrupt the JAK/STAT pathway resulting in transcriptional mis-regulation of associated genes and promoting tumor proliferation, migration and invasion. However, further study of *IL6ST* in a larger cohort is needed to understand its prognostic role in TNBC.

*ANKRD30A* showed significantly lower expression in TNBC compared to non-TNBC cases and its lower expression was associated with ER, PR status and tumor size; as well as worse RFS, suggesting a role in disease aggressiveness. Similar to the findings reported herein, another study have also found its downregulation in TNBC tissues (21, 22) and its expression was associated with ER status (23).Notably, *ANKRD30A* possesses an estrogen response element in the promoter region which may be regulated through estrogen receptor signaling (24), suggesting a possible mechanism for its low expression in TNBC. Additionally, the downregulation of Long Non-coding RNA LINC00993 (Long Intergenic Non-Protein Coding RNA 993) was significantly associated with the downregulated expression of the nearest coding gene, *ANKRD30A* in a microarray study of TNBC (21). It should be noted that many genomic regions can be co-regulated. Hence, the association may well be coincidental rather than causative. Interestingly, the lncRNA LINC00993 was identified to act as a tumor suppressor in TNBC which suppresses the growth of tumor cells both *in vivo* and *in vitro* (25). *ANKRD30A* is classed as a transcription factor due to the presence of bZIPsite and bipartite nuclear localization signal motif, hence it may be involved in regulating the expression of LINC00993. Thus, this implies that downregulation of *ANKRD30A* may have a significant role in tumor progression in TNBC.

*ANP32E* was significantly upregulated in TNBC compared to non-TNBC. Furthermore, its high expression was significantly associated with grade, the number of positive lymph nodes and worse RFS. *ANP32E* knockdown has been shown to inhibit the proliferation, migration and metastasis of breast cancer cells (26). It has also been shown to be highly expressed in primary breast cancers with a high propensity of metastasizing to the lungs (27). One study showed that *ANP32E* promotes G1/S progression by increasing the expression of E2F1, thus inducing proliferation in TNBC cells (28). The same study also showed that *ANP32E* is highly expressed at protein level in TNBC cases compared to non-TNBC cell lines and tumors. Taken together, these data support a role for *ANP32E* in cancer progression. However, further studies in a larger cohort are needed to understand its prognostic role in TNBC.

*DSC2* was significantly upregulated in TNBC compared to non-TNBC but was not associated with clinicopathological features or survival outcomes. Microarray gene expression analysis of 23 breast cancer metastases showed that the upregulation of *DSC2* expression may contribute to lung metastasis as a part of a 6-gene signature (27). Moreover, a study validated that the overexpression of five genes including *DSC2* worsens lung metastasis-free survival in geminin-overexpressing TNBC cells (29). In contrast, a reduction in *DSC2* expression has been identified in other cancer types such as colorectal, lung and esophageal squamous cell carcinoma (30–32). Conversely, a reduction in the expression of *DSC2* has been found in other cancers including colon cancer (33) and urothelial carcinoma tissues, where its downregulation was associated with rapid migration and invasion (34). Currently, there have been no other studies of *DSC2* expression and its role in TNBC.

Perhaps one of the most important findings was that when combined, the four-gene panel was strongly associated with RFS in TNBC, but not in hormone receptor positive breast cancers. Additionally, the four-gene panel provided a better survival curve discrimination when compared to each of the individual genes. The signature was further associated with RFS in 4/6 TNBC subtypes (**Figure 5**), indicating that this signature may be useful in predicting survival in the majority of TNBC cases. Interestingly, high expression of the gene panel was associated with better RFS in the LAR subtype, and worse RFS in the BL1, BL2 and mesenchymal subtypes. Given that xenografts developed from cell lines representative of these distinct subtypes show distinct responses to chemotherapeutic agents (14), the four gene panel defined in this study may have significant clinical utility, but these results would need to be validated in an independent cohort given the small sample size. Another key consideration is whether the mRNA expression of these four genes is correlated with their protein expression, the functional unit of the gene. There has been one published study showing that ANP32E is highly expressed at the mRNA and protein level in TNBC compared to non-TNBC tissues (28). However, for the other three genes, the correlation with protein expression has not been examined in breast cancer tissues to the best of our knowledge.

Taken together, these studies have verified the differential expression of *ANKRD30A, IL6ST, ANP32E* and *DSC2* in TNBC compared to non-TNBC and determined their association with clinical features and survival. Additionally, the four-gene panel may serve as a specific prognostic tool in TNBC management.

## Data Availability Statement

The raw data supporting the conclusions of this article will be made available by the authors, without undue reservation.

## Ethics Statement

The studies involving human participants were reviewed and approved by Hunter New England Human Research Ethics Committee. The ethics committee waived the requirement of written informed consent for participation.

## Author Contributions

KA-K: conceptualization. MP and KA-K: data collection, curation, and methodology. MP and KA-K: writing—original draft preparation. MP, RT, RS, KA-K: review and editing. RT, RS, and KA-K: supervision. RS and KA-K: funding acquisition. All authors contributed to the article and approved the submitted version.

## Funding

This work was funded by the National Breast Cancer Foundation and the Hunter Medical Research Institute, through donations from the Hunter Breast Cancer Foundation. MP was supported by a University Postgraduate Award. KA-K is supported by the Cancer Institute NSW (Career Development Fellowship; CDF181205).

## Conflict of Interest

The authors declare that the research was conducted in the absence of any commercial or financial relationships that could be construed as a potential conflict of interest.

## Publisher’s Note

All claims expressed in this article are solely those of the authors and do not necessarily represent those of their affiliated organizations, or those of the publisher, the editors and the reviewers. Any product that may be evaluated in this article, or claim that may be made by its manufacturer, is not guaranteed or endorsed by the publisher.
